# SARS-CoV-2 wastewater surveillance in a central Chinese metropolis: variant responses and correlation with clinical cases

**DOI:** 10.3389/fpubh.2026.1875582

**Published:** 2026-07-20

**Authors:** Xinye Zhang, Jie Zhang, Qiuyan Zhao, Xiaoyan Ma, Lingshuang Lv, Hongxia Ma, Shufang Zhang, Zhiwei Han

**Affiliations:** 1 Environmental Health Section, Henan Provincial Center for Disease Control and Prevention, Zhengzhou, China; 2 Institute of Environmental Health and Infection Control, Hunan Provincial Center for Disease Control and Prevention, Changsha, China; 3 Infectious Disease Prevention and Control Institute, Henan Provincial Key Laboratory of Infectious Disease Pathogens, Henan Province Center for Disease Control and Prevention, Zhengzhou, China

**Keywords:** China, COVID-19, physicochemical parameters, SARS-CoV-2, viral variant, wastewater

## Abstract

**Objective:**

This study analyzed SARS-CoV-2 surveillance results in the urban wastewater of Zhengzhou, central China, to explore its association with community COVID-19 incidence and evaluate its early warning performance across different SARS-CoV-2 variant epidemic phases.

**Methods:**

Weekly monitoring was conducted at five representative wastewater treatment plants from May 2024 to July 2025, yielding 325 samples. Viral loads of the ORF1ab and N genes were quantified using RT-qPCR, with statistical analyses performed included Spearman correlation, ANOVA, and cross-correlation function.

**Results:**

The two target genes showed high consistency and significant positive correlation with 14-day cumulative COVID-19 incidence. However, significant variant and marked heterogeneity was observed across variant periods and gene targets, with the N gene outperforming ORF1ab in stability. Wastewater viral signals preceded clinical cases by one week.

**Conclusion:**

These findings supported the application value of wastewater-based epidemiology in post-pandemic COVID-19 surveillance, indicate its potential early warning capacity, and reveal notable heterogeneity in surveillance performance across viral variants and target genes.

## 1 Introduction

Coronavirus disease 2019 (COVID-19), caused by the severe acute respiratory syndrome coronavirus 2 (SARS-CoV-2), has imposed an unprecedented, sustained burden on global public health and socioeconomic system (1). As of December 2025, a cumulative total of 779 million confirmed COVID-19 cases and more than 7 million deaths have been reported worldwide (2). In China, a major policy transition occurred on January 8, 2023, when COVID-19 management was downgraded from a Class A to a Class B infectious disease (3). Consequently, mass routine nucleic acid testing has been gradually phased out (4). Conventional clinical case-based surveillance systems are limited by inherent time lags (5), and substantial underreporting of asymptomatic and mild cases (6–8), reducing their ability to capture the real-time transmission dynamics (1, 9).

Wastewater-based epidemiology (WBE) has emerged as a complementary approach that enables community-level monitoring by detecting viral RNA shed into raw wastewater, thereby capturing both asymptomatic and mild infections (10–12). Benefiting from its cost-effectiveness, high timeliness, and population-wide coverage, WBE has been integrated into national SARS-CoV-2 surveillance frameworks in more than 70 countries and territories globally, and is now widely recognized as a cornerstone of community-level COVID-19 monitoring worldwide (1, 5, 10–12). The China Wastewater Surveillance System (CWSS), a pioneering national surveillance initiative, was officially launched in December 2022 (13, 14). This system conducts pathogen monitoring for high-priority infectious diseases, including SARS-CoV-2, poliovirus, and influenza virus, across 833 monitoring sites in 169 regions nationwide.

Extensive global and domestic studies have consistently demonstrated a significant positive correlations between SARS-CoV-2 RNA concentrations in wastewater and key clinical surveillance metrics, including community-reported case counts, COVID-19 hospitalizations, and outpatient fever clinic positivity rates (15–17). This robust and reproducible correlation underpins the core utility of wastewater-based epidemiology (WBE) for three critical public health applications: early warning of epidemic trends, unbiased estimation of community-level infection burden, and real-time monitoring of circulating viral variants (1, 17). In a landmark long-term, multisite study across England, which covered 65% of the national population and incorporated nearly 95,000 wastewater samples from 452 sampling locations, the median correlation coefficient between wastewater viral concentrations and community case counts reached 0.66 for wastewater treatment plant (WWTP) sites and 0.65 for sewer network site (SNS) sampling samples (17). Markedly, this stable association was consistent across catchments with widely varying population sizes, geographic characteristics, and socioeconomic statuses, highlighting the generalizability of WBE across diverse urban settings. Similar findings have been reported across continental Europe, with a nationwide surveillance study in the Czech Republic documenting significant positive correlations between wastewater SARS-CoV-2 RNA levels and community active case counts (Spearman *r* = 0.48–0.68) across all monitored regions (15). Concurrently, a nearly two-year continuous surveillance study in Changsha, central China, validated that the concentrations of SARS-CoV-2 RNA targeting the ORF1ab and N genes in wastewater exhibited Pearson correlation coefficients of 0.79 and 0.77, respectively, with locally reported COVID-19 cases, achieving a coefficient of determination (R2) of up to 0.63 in univariate linear regression models (18). This correlation has also been validated to be stable across different epidemic phases and variant dominance periods in multi-city studies across China, further supporting the reliability of WBE in domestic settings (13, 14, 18).

However, existing research has predominantly focused on correlation analyses during periods of large-scale epidemic transmission. There remains a critical paucity of studies evaluating the performance of WBE in the post-pandemic era, a setting characterized by low-level endemic transmission and the continuous emergence and replacement of SARS-CoV-2 variants (19–21). Furthermore, the quantitative dose–response relationship between wastewater viral concentrations and the actual number of infections in the community remains poorly characterized. Previous studies have documented that the strength of the correlation between WBE signals and clinical metrics varies significantly across epidemic phases and with the turnover of dominant viral variants (15, 18). Specifically, this correlation was markedly attenuated during the late phase of Omicron variant circulation, with a substantial increase in the heterogeneity of the quantitative relationship between wastewater viral concentrations and case counts across different catchments (11, 15, 17, 22). Meanwhile, considerable debate persists in the literature regarding the sensitivity of WBE in low-transmission settings, as well as the minimum community infection burden corresponding to the assay’s limit of detection (LOD) (23). Moreover, the development of robust quantitative models for infection burden estimation is hindered by a lack of long-term, multisite field validation data. In addition, wastewater viral concentrations are modulated by a multitude of factors, including wastewater physicochemical parameters, population-level viral shedding dynamics, and socioeconomic characteristics of the catchment population (11, 15, 24). Consequently, viral concentration alone is insufficient to accurately predict the scale of community infections.

In this study, we selected the urban core of Zhengzhou, a provincial capital city in central China, as the study area. Leveraging wastewater surveillance data, clinical COVID-19 surveillance data, and catchment population characteristic data collected between May 2024 and July 2025, we systematically characterized the dynamic relationship between SARS-CoV-2 RNA concentrations in wastewater and community-level COVID-19 incidence infection burden in the post-pandemic era. We further elucidated the heterogenous performance of WBE across successive epidemic waves driven by distinct SARS-CoV-2 variants, as well as the modulating effects of wastewater physicochemical parameters on viral detection. On this basis, evaluate the epidemic early warning capability of WBE, quantify the differences in the correlation between wastewater data and clinical data during the three waves of Omicron outbreaks as well as the discrepancies in monitoring performance across five different urban functional zones, so as to provide a scientific basis for optimizing the wastewater-based surveillance system for COVID-19 in the post-pandemic era.

## 2 Materials and methods

### 2.1 Study location

The study was conducted in Zhengzhou, the capital city of Henan Province in central China. Zhengzhou (east longitude 112°42′-114°14′, north latitude 34°16′-34°58′) was one of the eight ancient capitals of China, with a land area of 7,567.2 km^2^ and a population exceeding 13 million. The city has achieved an urbanization rate of 80.0%, resulting in the second-highest population density among all provincial capitals in China. Zhengzhou experiences a warm temperate continental monsoon climate. Geographically, it is situated in the transition district between the middle and lower reaches of the Yellow River, straddling both the Yellow River and Huaihe River basins. The Jialu River serves as the primary inland waterway, traversing the city’s urban core.

This study focused on five representative wastewater treatment plants (WWTPs) situated in distinct functional districts of Zhengzhou: WWTP-A in the southwestern central district, a major commercial hub; WWTP-B in the northern ecological residential district along the Yellow River; WWTP-C in the historic urban core, characterized by high population density; WWTP-D in the eastern new district, serving as the modern financial and administrative center; WWTP-E in the western high-tech industrial and innovation cluster. Collectively, these facilities serve Zhengzhou’s primary urban functional areas, catering to a combined population of approximately 4.3 million. Wastewater surveillance was conducted from May 6, 2024 (Week 19) to July 28, 2025 (Week 31).

### 2.2 Laboratory analysis

Wastewater sampling was conducted at the main influent channel of each WWTP. Flow-proportional composite samples were collected over a 24-h period using automated refrigerated samplers, which accumulated 100 mL aliquots hourly to yield a total volume of 2,400 mL. Following the collection cycle, the hourly aliquots were homogenized to form a representative composite sample. For SARS-CoV-2 analysis, a 500 mL sub-sample was immediately stored at 4 °C and transported to the laboratory within 2 h to ensure viral integrity. Sampling was performed weekly on Mondays. Concurrently, operational data including daily wastewater treatment volume (WTV), influent temperature, total suspended solids (TSS), chemical oxygen demand (CODCr), pH, and ammonia nitrogen were retrieved from the online monitoring equipment records of the wastewater treatment plant.

### 2.3 Inactivation, nucleic acid extraction, and RT-qPCR detection

The sealed sample bottles were completely immersed in a 60 °C water bath for 30 min to inactivate SARS-CoV-2 in wastewater samples. Viral enrichment, concentration, and nucleic acid detection were performed in accordance with the National Health Commission of China Standard WS/T 799–2022: Method for enrichment and nucleic acid detection of SARS-CoV-2 in sewage (25). Briefly, viruses were concentrated via polyethylene glycol (PEG) precipitation, followed by total nucleic acid extraction. Detection was performed using a dual-target reverse transcription quantitative polymerase chain reaction (RT-qPCR) assay targeting the open reading frame 1ab (ORF1ab) and nucleocapsid protein (N) genes of SARS-CoV-2 (26).

For quantification, separate standard curves were established for the ORF1ab and N genes in each run using certified SARS-CoV-2 RNA standards with at least six serial dilutions (minimum five valid points). The lowest concentration point was set slightly above the kit’s limit of detection (LOD). Amplification was conducted on a real-time PCR system, and data validity was strictly controlled: standard curves were accepted only if the amplification efficiency ranged from 90 to 110% and the coefficient of determination (R_2_) was ≥ 0.99. For each sample, the mean Ct value of technical replicates for each individual target gene was interpolated into the standard curve to calculate the SARS-CoV-2 concentration in samples, expressed in copies per milliliter (copies/mL).

### 2.4 Data processing

The service catchment of each WWTP was delineated based on geographic information system (GIS) data of the municipal sewer network, served populations were estimated by summing community-level census counts within each drainage boundary. The daily COVID-19 cases data were sourced from the China Disease Prevention and Control Information System. To align with the sampling dates, we calculated the 14-day cumulative incidence for each monitoring site. Specifically, for each daily case count, a 14-day sliding window was constructed, spanning 10 days prior to, the day of, and 3 days following the sampling event. The 14-day sliding window was designed based on the incubation period (2–14 days) and viral shedding cycle of SARS-CoV-2 Omicron subvariants (15, 27, 28). The total case count within this window (cases_14d) was summed, and the 14-day cumulative incidence (per 100,000 population) was computed as follows:


Incidence_14d=(cases_14d/population)×100,000.

Epidemiological data on circulating SARS-CoV-2 variants in the human population were obtained from the China Disease Prevention and Control Information System. Temporal analysis of variant dominance revealed a sequential shift in prevalent lineages: the JN.1 series predominated from May to September 2024, followed by the XDV series, which remained dominant until April 2025. Subsequently, the NB.1.8.1 lineage emerged as the prevailing strain from May to July 2025 onwards.

To quantify viral shedding dynamics independent of hydraulic fluctuations and population size, raw wastewater viral concentrations were normalized to estimate the per capita daily viral load. The calculation procedure involved three steps:

The total daily viral load (Total_Virusgene, copies/day) for each WWTP was calculated by multiplying the daily wastewater treatment volume (WTV) by the measured viral concentration(Cgene), with appropriate unit conversions:


Total_Virusgene=WTV×1,010×Cgene.

Where WTV was the daily wastewater treatment volume (expressed in 10^4^ m^3^/day);

Cgene represented the concentrations of SARS-CoV-2 RNA (ORF1ab, N) in wastewater.

(copies/mL).

To account for variations in the service population across different WWTPs, the total daily viral load was normalized by the served population size (Population) to obtain the per capita daily viral load(Vgene):


Vgene=Total_Virusgene/Population.

Where Vgene is expressed in copies·day-1·person-1, and the gene represented the ORF1ab and N, respectively. Population represented the number of residents served by the specific WWTPs.

### 2.5 Statistical analysis

Data collation and analysis were carried out using R software (version 4.4.2). Spearman correlation analysis was performed to assess the correlations between SARS-CoV-2 RNA concentrations in wastewater and 14-day cumulative COVID-19 incidence, as well as wastewater physicochemical parameters (11, 15). For each site, one-way analysis of variance (ANOVA) was performed to compare unit viral load across different variant periods. For sites with a significant ANOVA result (*p* < 0.05), Tukey’s honestly significant difference (HSD) test was applied for post-hoc pairwise comparisons. To evaluate the predictive potential, cross-correlation function (CCF) analysis was performed between the per capita daily SARS-CoV-2 RNA(ORF1ab, N) viral load and the 14-day cumulative COVID-19 incidence at each monitoring site. A maximum lag of 4 was set, with each lag representing 7 days, to determine the optimal lag period for predicting clinical cases using wastewater viral load (13, 16). The Augmented Dickey-Fuller (ADF) test was performed to verify the stationarity of the time series data prior to CCF analysis (29), and all sequences were stationary after differencing if needed (*p* < 0.05).

## 3 Results

### 3.1 Sewage parameter information

A total of 325 samples were analyzed, with 65 samples collected from each of the five WWTPs. The median values (P_25_, P_75_) of wastewater-related parameters for these five WWTPs are shown in Table 1. These five WWTPs served a population ranging from 100,000 to 1.85 million, with WWTP-B being the largest in scale. Regarding physicochemical parameters, the median inlet water temperature across the five WWTPs ranged from 23.3–24.9 °C, total suspended solids (TSS) ranged from 231.0–361.0 mg/L, and chemical oxygen demand (CODCr) ranged from 246.0–397.0 mg/L. Ammonia nitrogen concentrations ranged from 24.1–47.4 mg/L, with pH values consistently falling within the neutral to weakly alkaline range (7.2–7.4).

**Table 1:** Wastewater parameter information for the five WWTPs monitored [25th–75th percentile (P_25_, P_75_)].

Variables	WWTP-A	WWTP-B	WWTP-C	WWTP-D	WWTP-E
WTV (10,000 tons/day)	3.3 (3.0, 3.4)	63.5 (61.6, 66.0)	9.0 (7.5, 9.9)	13.1 (11.2, 14.5)	16.6 (15.8, 17.3)
Inlet water temperature (°C)	24.9 (20.1, 28.1)	23.7 (18.1, 26.5)	24.3 (18.6, 27.8)	23.3 (18.4, 26.2)	24.1 (18.7, 27.5)
Total suspended solids (mg/L)	296.0 (225.5, 363.0)	231.0 (173.0, 262.0)	268.0 (195.0, 319.0)	278.0 (251.5, 333.0)	361.0 (304.5, 414.0)
CODCr (mg/L)	309.5 (254.5, 390.0)	246.0 (186.0, 340.0)	296.0 (226.0, 373.0)	349.0 (295.5, 400.0)	397.0 (343.0, 488.0)
pH	7.4 (7.3, 7.5)	7.3 (7.2, 7.4)	7.4 (7.3, 7.4)	7.3 (7.2, 7.4)	7.2 (7.1, 7.4)
Ammonia nitrogen (mg/L)	24.1 (18.2, 28.0)	27.4 (24.3, 29.5)	42.2 (35.3, 45.6)	40.6 (37.1, 44.6)	47.4 (44.0, 51.8)
Population served	260,000	1,850,000	100,000	1,200,000	880,000

### 3.2 Relationship between SARS-CoV-2 RNA concentration in wastewater and the reported number of COVID-19 cases

**Figure 1:**
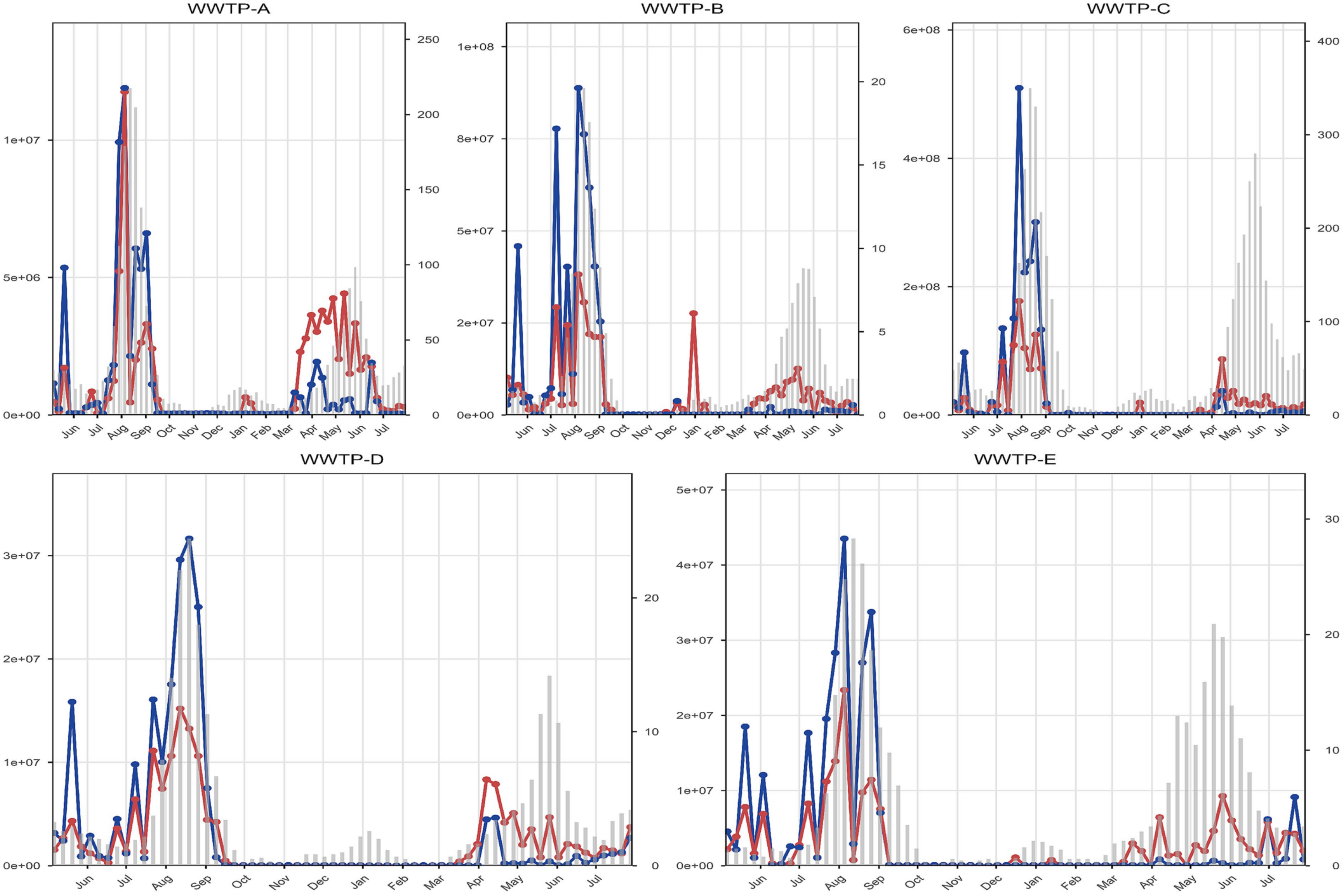
Temporal trends of per capita daily SARS-CoV-2 viral load (ORF1ab and N genes) and 14-day cumulative COVID-19 incidence across five WWTPs. The blue line graph represented per capita daily viral load gene ORF1ab, while the red line graph represented per capita daily viral load gene N. Gray bars represented 14-day cumulative incidence. The horizontal axis represented time from May 6, 2024 to July 28, 2025. Y-axis (left) -per capita daily SARS-CoV-2 RNA viral load, secondary Y-axis (right)-14-day cumulative COVID-19 incidence.

Temporal trends in the per capita daily viral load (ORF1ab, N) in wastewater were highly consistent with the 14-day cumulative COVID-19 incidence (Figure 1), with distinct viral load peaks aligning with the three dominant variant waves (JN.1, XDV, and NB.1.8.1). Elevated per capita daily viral loads were observed during the JN.1 variant period, coinciding with a surge in 14-day cumulative incidence. During the XDV variant wave, viral concentrations remained persistently low, mirroring sustained low community transmission. A moderate resurgence in viral load was recorded during the NB.1.8.1 period, which closely tracked the rise in cumulative incidence. Notably, the temporal patterns of the target genes ORF1ab and N were nearly identical across all study periods, validating the reliability of wastewater-based surveillance for COVID-19. Substantial cross-site differences in per capita viral loads align with the population density and mobility characteristics of each functional zone.

**Figure 2:**
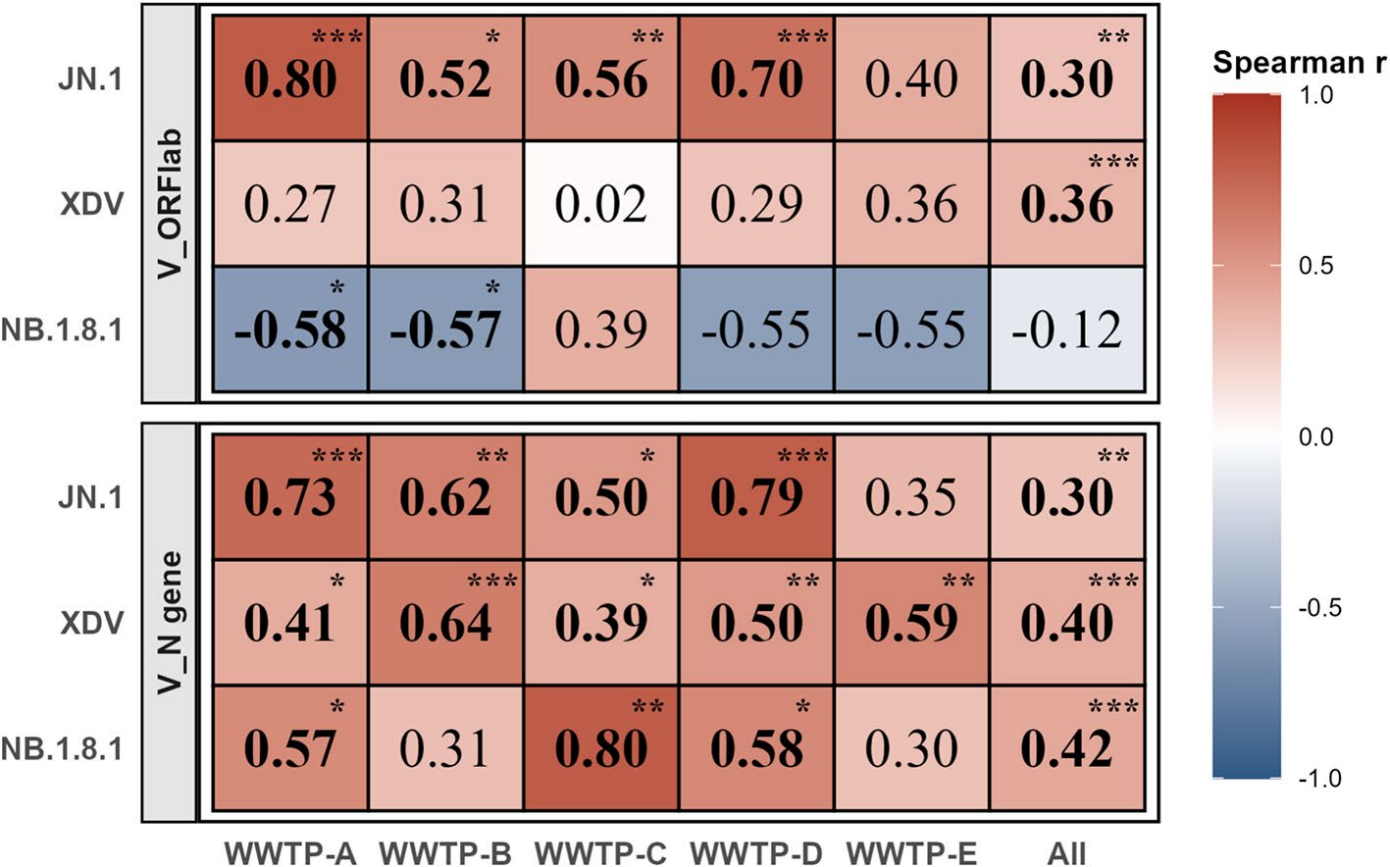
Spearman Correlations between per capita daily SARS-CoV-2 RNA(ORF1ab, N) viral load in wastewater and 14-day cumulative COVID-19 incidence across different SARS-CoV-2 variant periods. Significance levels: *** *p* < 0.001, ** *p* < 0.01, * *p* < 0.05.

During the JN.1 variant wave, significant positive correlations were observed between per capita daily viral load (ORF1ab, N) and 14-day cumulative incidence at all monitored wastewater treatment plants (WWTPs) except WWTP-E, with Spearman correlation coefficients ranging from 0.30 to 0.80 (*p* < 0.05). Moderate but predominantly significant positive correlations persisted during the XDV variant period. Notably, divergent patterns emerged during the NB.1.8.1 wave: the gene N remained positively correlated with incidence at most sites, while the target gene ORF1ab exhibited significant negative correlations at WWTP-A and WWTP-B, suggesting variant-specific differences in viral shedding dynamics (Figure 2).

**Figure 3:**
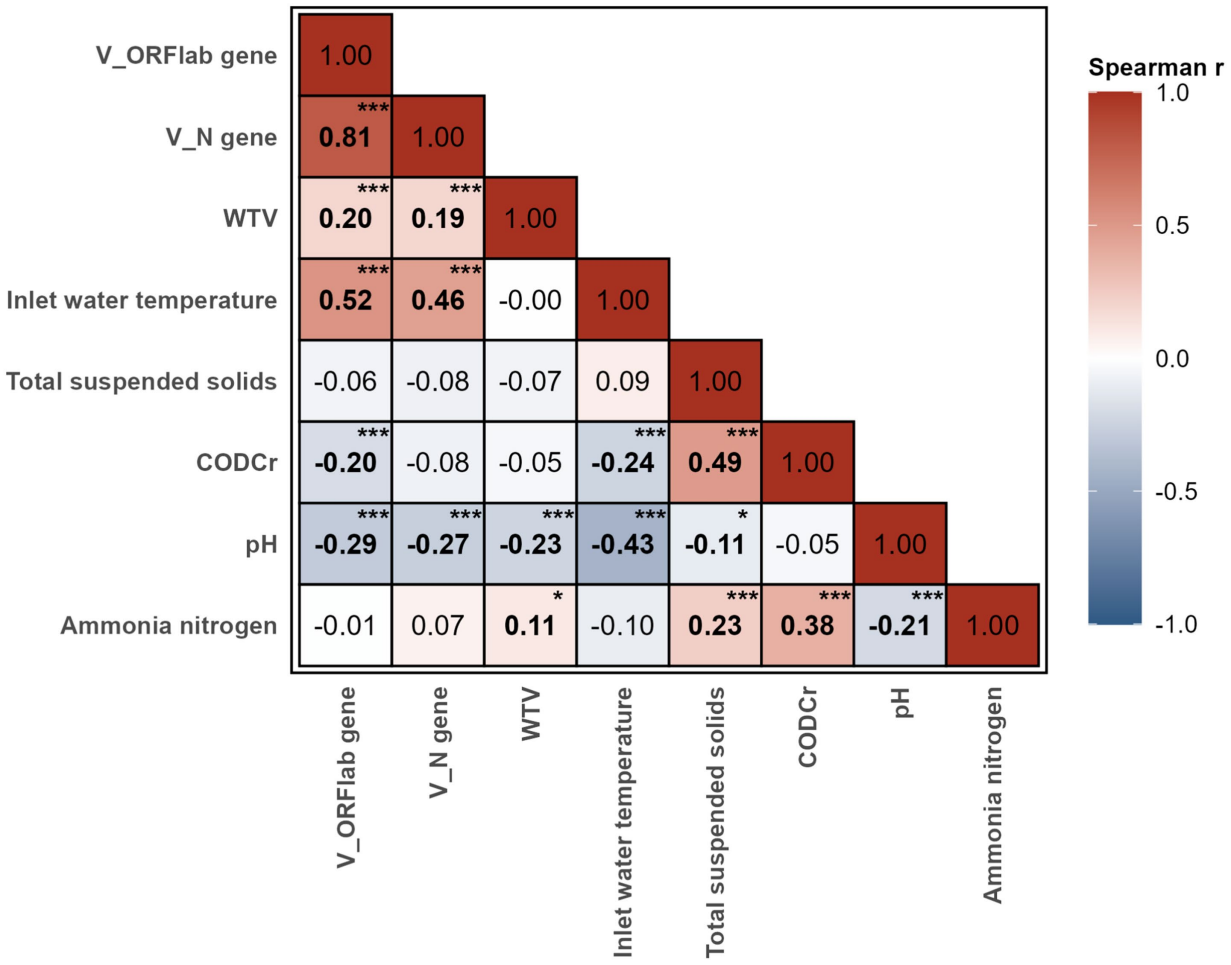
Spearman Correlations between per capita daily SARS-CoV-2 RNA(ORF1ab, N) viral load and physicochemical indicators in wastewater. Significance levels: *** *p* < 0.001, ** *p* < 0.01, * *p* < 0.05.

Across the study period, per capita daily viral load for the target gene ORF1ab and N were significantly positively correlated with inlet water temperature (*r* = 0.52 and 0.46, respectively, *p* < 0.001) and significantly negatively correlated with pH (*r* = −0.29 and −0.27, respectively, *p* < 0.001). After controlling for seasonal confounding via partial correlation analysis, influent temperature remained independently and positively associated with viral loads, while the association with pH was attenuated. No meaningful correlations were observed between viral load and other wastewater physicochemical parameters, including total suspended solids, CODCr, and ammonia nitrogen (Figure 3).

### 3.3 Cross-correlation analysis of wastewater SARS-CoV-2 RNA and COVID-19 community incidence

**Figure 4:**
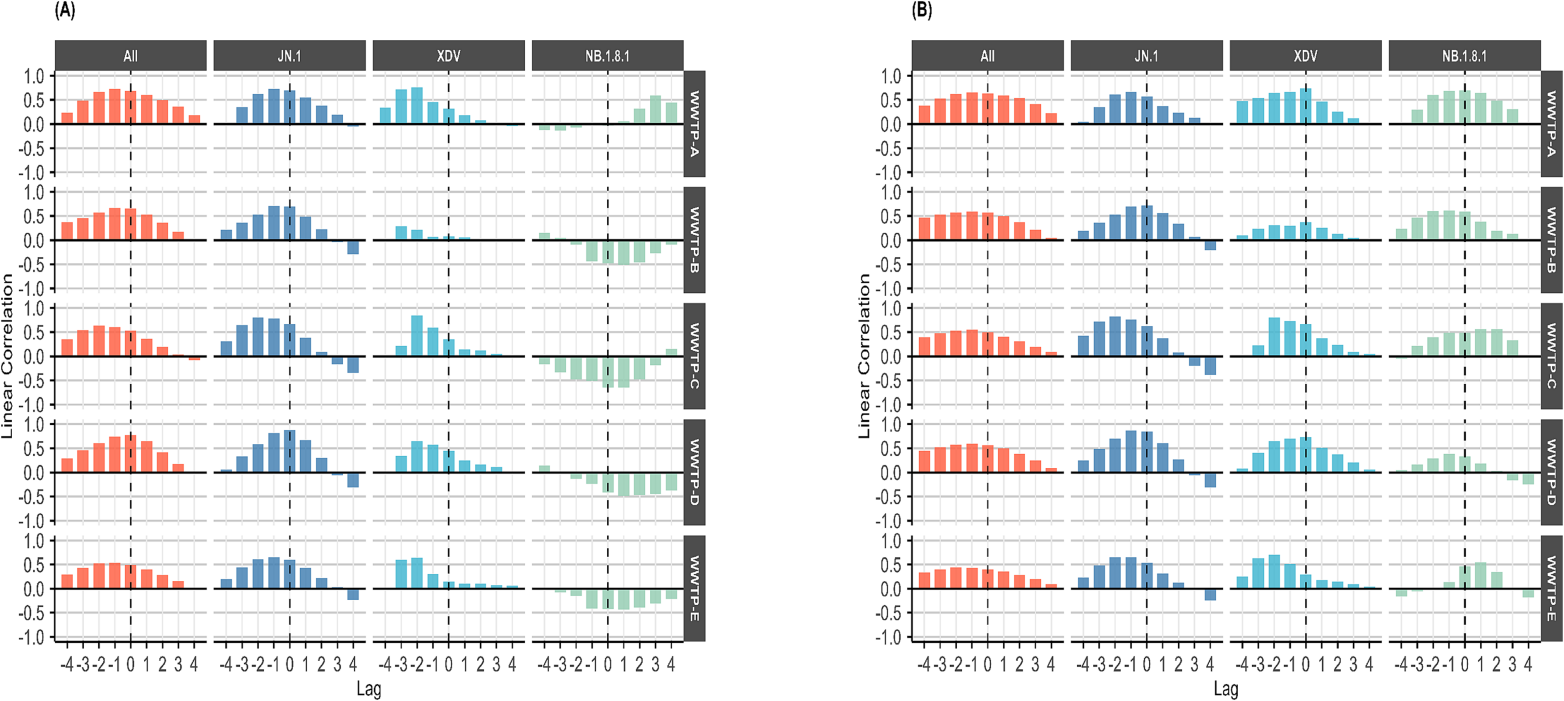
Cross-correlation between per capita daily viral load SARS-CoV-2 RNA **(A)** ORF1ab, and **(B)** N and the 14-day cumulative COVID-19 incidence at five WWTPs during the study period.

This study analyzed the cross-correlation between the per capita daily viral loads in wastewater and the 14-day cumulative COVID-19 incidence for the five WWTPs (Figure 4). Stratified analyses were further conducted according to three epidemic phases dominated by SARS-CoV-2 variants JN.1, XDV, and NB.1.8.1, respectively. Overall, the per capita daily viral loads of SARS-CoV-2 in wastewater exhibited a significant positive correlation with the 14-day cumulative COVID-19 incidence in the service-area communities. The correlations were relatively stronger at lag = 0 and −1 (where lag = −1 indicate that wastewater viral loads preceded the incidence data by one week). Heterogeneity existed in the correlations across different variant epidemic phases. During the JN.1 epidemic phase, the two target genes (ORF1ab, N) exhibited consistent trends in different WWTPs. During the NB.1.8.1 epidemic phase, the target gene ORF1ab showed an essentially negative correlation with incidence rates, whereas the target gene N still maintained a generally positive correlation with incidence rates. Sensitivity analyses were conducted using disease onset time windows of 14 days and 7 days prior to the sampling date, respectively, with consistently robust results obtained.

## 4 Discussion

This study constructed a long-term wastewater surveillance dataset from May 2024 to July 2025 and investigated five wastewater treatment plants (WWTPs) covering distinct functional districts in Zhengzhou, China. We systematically characterized the temporal dynamics of SARS-CoV-2 RNA loads in wastewater and their associations with 14-day cumulative COVID-19 incidence at the community level. We further quantified variant-dependent correlations, identified key wastewater physicochemical parameters influencing viral detection, and evaluated the early warning capacity of Wastewater-based epidemiology (WBE). This study not only confirmed the wastewater-clinical association reported in the Czech Republic (15) and several Chinese cities (13, 18, 30), but also, for the first time under sequential Omicron subvariant epidemics, revealed divergent detection performance between the ORF1ab and N genes as variants succeed each other, as well as monitoring heterogeneity across different functional zones within the same city.

Wastewater concentrations of per capita daily SARS-CoV-2 RNA (ORF1ab, N) exhibited consistent temporal trends with 14-day cumulative COVID-19 incidence across the study period, which were similar to previous results (31, 32). Peaks in viral load coincided with epidemic waves dominated by three major variants: JN.1, XDV, and NB.1.8.1. The parallel trends of ORF1ab and N throughout monitoring confirmed the robustness of WBE in reflecting community transmission dynamics (33, 34). These observations align with reports from Lv et al., who documented strong positive correlations between wastewater ORF1ab/N gene concentrations and clinical cases (Spearman *r* = 0.79 and 0.77, respectively; *p* < 0.05) (18), and Sovová et al., who reported significant synchrony between wastewater SARS-CoV-2 RNA and community active cases (*r* = 0.48–0.68) (15).

Notable heterogeneity in correlations was observed across variant periods. During JN.1 dominance, per capita viral loads of both gene targets correlated positively with 14-day cumulative incidence at four of five WWTPs (Spearman *r* = 0.30–0.80, *p* < 0.05), representing the strongest and most stable associations. Moderate positive correlations persisted during XDV circulation. In contrast, NB.1.8.1 (a sixth-generation Omicron subvariant) introduced target-dependent divergence: N gene remained positively correlated with incidence at most sites, whereas ORF1ab showed significant negative correlations at WWTP-A and WWTP-B. Similar shifts in correlation strength and direction across successive variants have been widely reported in national and regional WBE programs (35–38).

We observed significant associations between wastewater physicochemical parameters and SARS-CoV-2 RNA concentrations. After controlling for seasonal confounding via partial correlation analysis, influent temperature remained independently and positively associated with viral loads, while the association with pH was notably attenuated. The temperature-virus association may be driven by direct effects on RNA degradation kinetics. In contrast, pH effects are more susceptible to seasonal confounding.

Cross-correlation function (CCF) analysis revealed peak associations between wastewater per capita viral load and 14-day cumulative incidence at lags of 0 and −1, suggesting a general leading trend of WBE signals roughly one week ahead of reported clinical cases. Warning performance was strongest during JN.1 and XDV circulation but diminished under NB.1.8.1 dominance, likely modulated by local demography, mobility patterns, and infrastructure. Notably, during NB.1.8.1 circulation, the target gene ORF1ab inversely correlated with reported incidence in WWTP-C, while N gene maintained positive correlations across most sites. These results highlight urban functional zoning and population structure as critical confounders that require context-specific calibration for reliable public health surveillance (39–41).

Several limitations warrant consideration. First, the inherent complexity of the wastewater matrix introduced intrinsic uncertainty into RNA quantification. We neither applied normalization using fecal indicator viruses such as pepper mild mottle virus (PMMoV), nor explicitly modeled the survival, persistence and degradation kinetics of SARS-CoV-2 in sewage, which may have introduced bias in the estimation of absolute viral loads. Second, whole-genome sequencing was only performed on positive samples with Ct ≤ 34, yielding a limited number of high-quality sequences with uneven temporal coverage. This mechanistic explanation therefore remains a working hypothesis. Third, the cross-correlation function (CCF) analysis conducted using weekly sampling data reduced the temporal resolution of the analysis; therefore, caution should still be exercised when interpreting specific lag days. Future studies with higher sampling frequencies would have further enhance the accuracy of near-real-time epidemic early warning systems. Fourth, COVID-19 cases were geocoded by residential address without adjustment for cross-district commuting flows, and the design-based service population estimates only approximated the actual dynamic population within each sewershed, which might have caused mild misalignment between case attribution and WWTP catchment boundaries.

## 5 Conclusion

In summary, SARS-CoV-2 RNA loads in urban wastewater were strongly correlated with community COVID-19 incidence, and showed a general leading trend of roughly one week ahead of clinical case surges. Wastewater temperature was independently associated with viral detection efficiency, while the effect of pH was partially confounded by seasonal variation. Viral evolution drove pronounced variant-dependent differences in surveillance performance, with notable discrepancies in the correlation between the two target genes (ORF1ab and N gene) and reported incidence across monitoring sites. With a spatially representative sampling design covering five core urban functional zones serving approximately 4.3 million residents, this study effectively captures intra-city transmission heterogeneity shaped by urban functional zoning and population structure. Direct extrapolation of these findings to regions with distinct demographic or infrastructural characteristics required further local validation.

## Data Availability

The raw data supporting the conclusions of this article will be made available by the authors, without undue reservation.
